# Mechanical strain exacerbates *Pseudomonas* infection in an organoid-based pneumonia-on-a-chip model

**DOI:** 10.1172/JCI192454

**Published:** 2025-11-18

**Authors:** Karen Hoffmann, Ulrike Behrendt, Peter Pennitz, Holger Kirsten, Jessica Pohl, Elena Lopez-Rodriguez, Chantal Weissfuss, Jens Kollmeier, Mario Tönnies, Sebastian Brill, Konrad Steinestel, Martin Witzenrath, Werner Wenzel, Christian Zobel, Geraldine Nouailles

**Affiliations:** 1 Charité — Universitätsmedizin Berlin, Department of Infectious Diseases, Respiratory Medicine and Critical Care, Berlin, Germany.; 2 Bundeswehr Hospital Berlin, Department of Internal Medicine, Berlin, Germany.; 3 Universität Leipzig, ScaDS.AI, Institute for Medical Informatics, Statistics, and Epidemiology, Leipzig, Germany.; 4 Charité — Universitätsmedizin Berlin, Institute of Functional Anatomy, Berlin, Germany.; 5 Respiratory Diseases Clinic Heckeshorn, Helios Klinikum Emil von Behring GmbH, Berlin, Germany.; 6 Bundeswehr Hospital Ulm, Department for Thoracic-Surgery and Institute of Pathology and Molecular Pathology, Ulm, Germany.; 7 German Center for Lung Research (DZL), Berlin, Germany.; 8 Bundeswehr Hospital Berlin, Department of Microbiology and Hospital Hygiene, Berlin, Germany.

**Keywords:** Infectious disease, Pulmonology, Adult stem cells, Bacterial infections

## Abstract

Using an advanced human lung-on-a-chip model, this study shows that increased mechanical strain can make the lungs more vulnerable to harmful bacteria such as *Pseudomonas aeruginosa*.

**To the Editor:** *Pseudomonas aeruginosa* ventilator-associated pneumonia (VAP) is one of the most frequent nosocomial infections in mechanically ventilated ICU patients, with mortality rates, ranging from 24% to 76% (1), worsened by multidrug resistance. Still, VAP pathophysiology remains unclear, partly due to limited model systems.

Today, organotypic models, like the Emulate alveolus chip, can replicate alveolar-capillary physiology, including air-liquid interface (ALI), vascular flow, and mechanical forces. Here, we advanced the alveolus chip into a *Pseudomonas* pneumonia-on-a-chip (POC) model using human primary pulmonary microvascular endothelial cells and human alveolar epithelial cells of different origin (Figure 1A).

As barrier integrity and terminal differentiation into alveolar type 2 (AT2) and alveolar type 1 (AT1) cells are essential for lung function in vivo, we assessed these features in commercial primary alveolar epithelial cells (CPAECs) and organoid-derived alveolar epithelial cells (ODAECs) cultured on chips. We also examined the impact of cyclic deformations at a respiratory-like frequency (0.25 Hz) with varying intensities, mimicking either physiological (5%) (2) or hyperphysiological (10%) mechanical strain (3).

All cell types formed a confluent layer with immunofluorescence and apparent permeability (P_app_) measurements confirming tight barrier formations (Supplemental Figure 1, A and B; supplemental material available online with this article; https://doi.org/10.1172/JCI192454DS1). Yet, P_app_ was slightly increased in 10% stretched ODAECs, suggesting barrier impairment by hyperphysiological stretch.

LysoTracker staining, which marks surfactant-producing lamellar bodies, revealed larger and brighter structures in ODAECs compared with CPAECs (Figure 1B and Supplemental Figure 1C). Transmission electron microscopy (Figure 1C and Supplemental Figure 1D) further confirmed mature lamellar bodies in ODAECs, while CPAECs contained only lipid droplets, indicating that surfactant-producing AT2 cells were exclusive to ODAEC chips.

Moreover, while CPAECs stained positive for AT2 marker HTII-280 (Supplemental Figure 2A), mRNA expression of *SFTPC*, *SFTPB*, and *ABCA3* was only detectable for ODAECs (Figure 1E). Intriguingly, evidence of AT1-like cells in ODAECs was supported by immunofluorescence for AT1 markers HTI-56 and RAGE/AGER (Figure 1D and Supplemental Figure 2B) as well as mRNA expression of *AGER*, *HOPX*, and, to a lesser extent, *PDPN* (Figure 1E).

To elucidate factors influencing AT1 differentiation, we cultured ODAECs under static conditions and without endothelial cells on chip, as well as in Transwells under liquid-liquid and ALI conditions.

Surprisingly, while AT1 marker expression increased across all conditions, comparing the 2D with the organoid cultures, neither ALI nor stretch or the presence of endothelial cells seemed to affect AT1 differentiation, and it had minimal impact on AT2 differentiation (Supplemental Figure 2, C and D).

Single-cell RNA sequencing of stretched ODAECs and CPAECs, integrated with alveolar organoid data (4), confirmed the presence of AT1-like cells and fewer AT2 cells in ODAECs (Figure 1F and Supplemental Figure 3, A and B). In contrast, CPAECs remained in an intermediate basaloid cell state, failing to differentiate into AT1 or AT2 cells on chip.

Differential gene expression analysis between 10% and 5% stretched cells revealed more downregulated than upregulated genes across all major cell types (Figure 1G and Supplemental Figure 3C). This indicates that increased mechanical strain broadly suppresses gene expression in both epithelial and endothelial populations.

Having established that ODAECs exhibit superior differentiation capacity and that increased stretch substantially alters gene expression, we next introduced *Pseudomonas* (strain PAO1-GFP) as a causative agent of VAP. At 12 hours postinfection (hpi), CFUs, permeability, and cytokine levels were assessed. CFUs in the vascular channel and P_app_ were higher in 10% compared with 5% stretched ODAEC chips (Figure 1, H and I, and Supplemental Figure 4, A and B), indicating that mechanical strain exacerbates *Pseudomonas* infection in the alveolus. CPAECs showed similar but less pronounced effects, potentially due to limited differentiation or higher cell densities, as observed via imaging (Figure 1, B and D). Measurements of proinflammatory cytokines IL-6 and IL-8 confirmed strong immune activation in both ODAECs and CPAECs (Supplemental Figure 4C). Notably, lower cytokine levels were detected under 10% compared with 5% stretch, suggesting that increased strain modulates immune reactivity. Consistently, IL-6 and IL-8 transcripts were also downregulated in 10% stretched AT2 cells (Supplemental Figure 3C).

We hereby present what we believe to be the first functional *Pseudomonas* POC and VAP-on-a-chip model that replicates key features of disease, including enhanced mechanical strain. Notably, increased cellular stretch intensified *Pseudomonas* infection, leading to greater barrier disruption and bacterial translocation, demonstrating the chip’s ability to simulate early stages of VAP.

Additionally, ODAECs showed improved differentiation ability on chip, giving rise to both AT2 and AT1-like cells. Future studies should further explore how mechanical strain affects gene expression and contributes to infection severity and barrier breakdown. Going forward, we aim to investigate how this model captures pathogen colonization and virulence mechanisms — critical processes in the progression of pneumonia from initially opportunistic infections.

For detailed methods, information regarding sex as a biological variable, statistics, study approval, data availability, author contributions, and acknowledgments, see the supplemental materials.

## Funding support

- Federal Office of Bundeswehr Equipment, Information Technology and In-Service Support (BAAINBw) grant E/U2ED/PD014/OF550.
- Bundeswehr Medical Academy SoFo 39K4-S-452124.
- Deutsche Forschungsgemeinschaft (German Research Foundation), project ID 431232613 – SFB 1449.
- Federal Ministry of Research, Technology and Space (BMFTR), within project “Center for Scalable Data Analytics and Artificial Intelligence (ScaDS.AI) Dresden/Leipzig,” grant 01IS18026B.

## Supplementary Material

Supplemental data

Supporting data values

## Figures and Tables

**Figure 1:**
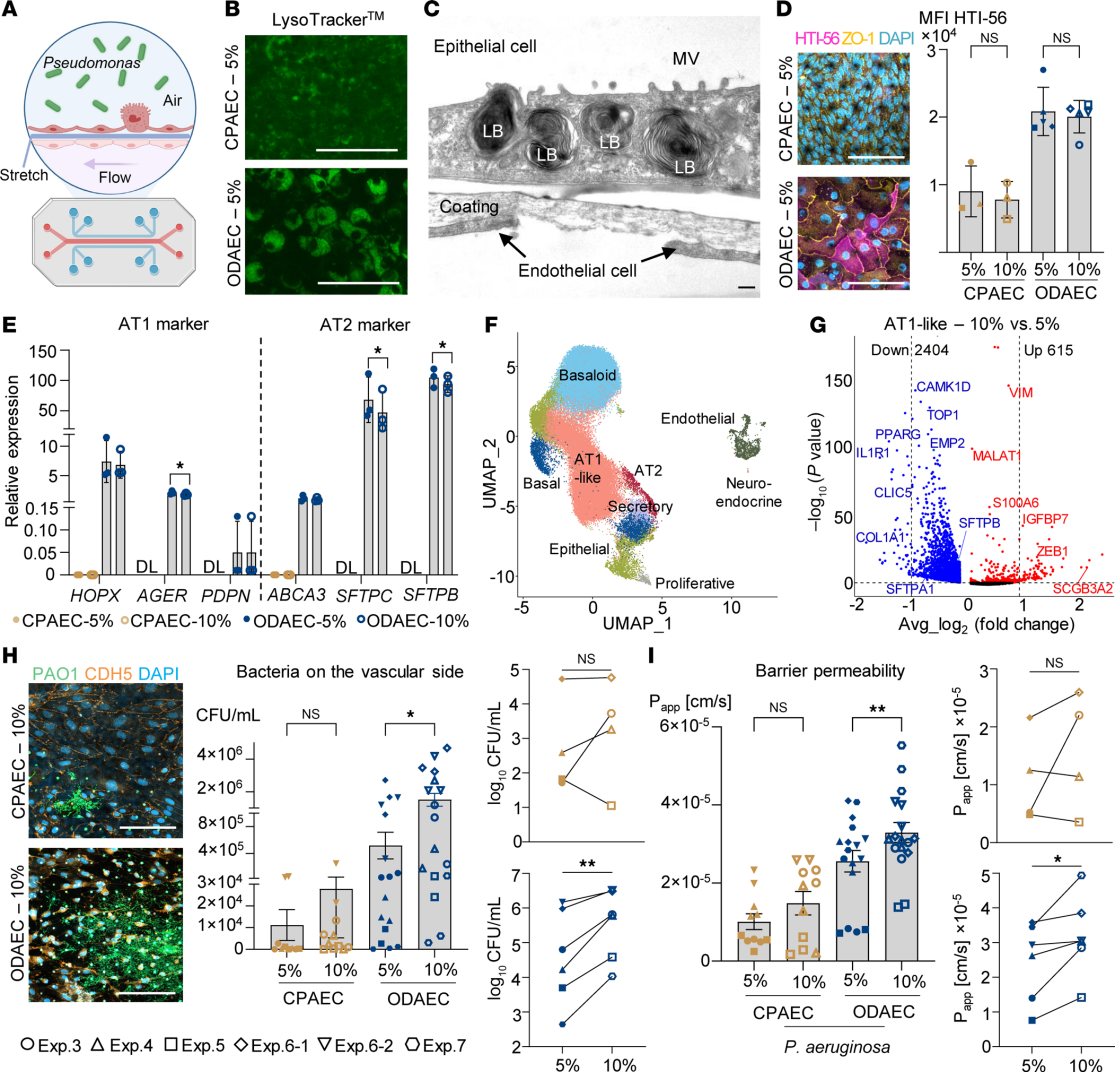
Organoid-derived alveolar cells differentiate on a stretchable microfluidic device and show force-dependent susceptibility to *Pseudomonas* infection. (**A**) Experimental outline. (**B**) Live-cell staining with LysoTracker. Scale bar: 100 μm. (**C**) Transmission electron micrograph of an ODAEC chip. Scale bar: 250 nm. LB, lamellar body; MV, microvilli. (**D**) Immunofluorescence staining of AT1 marker HTI-56 (magenta) and tight junction marker ZO-1 (yellow). Scale bar: 100 μm. Mean fluorescence intensities (MFI). Different symbols indicate independent experiments. Two-tailed paired *t* tests (*P* < 0.05). (**E**) Relative expression of AT1 and AT2 marker genes in CPAECs and ODAECs, normalized to GAPDH, measured by qPCR. Data are shown as the mean ± SEM, with data points from 3 independent experiments. DL, detection limit. One-way ANOVA (*P* < 0.05). (**F**) Uniform manifold approximation and projection (UMAP) plot of identified cell populations estimated from single-cell RNA-sequencing data from 1 CPAEC and 1 ODAEC donor each on-a-chip (5% and 10% stretch) integrated with a previously generated dataset of alveolar organoids (ref. 4; accession GSE197949). (**G**) Volcano plot of differentially expressed genes in 10% vs. 5% stretched AT1-like cells from the chip. (**H**) Immunofluorescence images of *Pseudomonas* (green) in the vascular channel (VE-Cadherin, orange) and quantification of bacterial load (CFU/mL) at 12 hpi. Scale bar: 150 μm (**I**) Apparent permeability (P_app_) at 12 hpi. (**H** and **I**) In bar graphs, data are shown as the mean ± SEM, with data points of all chip replicates over 4 independent experiments and 3 different donors for CPAECs and ODAECs. One-way ANOVA. In paired line plots, data are shown as the mean of chip replicates of each independent experiment. Two-tailed paired *t* tests (*P < 0.05, **P < 0.005).

## Version 1 11/18/2025

In-Press Preview

## Version 2 01/16/2026

Electronic publication
