# Supplementary material for: Mechanical strain exacerbates Pseudomonas infection in an organoid-based pneumonia-on-a-chip model
Source: J Clin Invest. 2025 Nov 18;136(2):e192454. doi: 10.1172/JCI192454 (PMC12807462; doi:10.1172/JCI192454)
Supplement: Supplemental data [file jci-136-192454-s254.pdf]

Supplementary Material

Mechanical strain exacerbates *Pseudomonas* infection in an organoid-based pneumonia-on-a-chip model

Karen Hoffmann, Ulrike Behrendt, Peter Pennitz, Holger Kirsten, Jessica Pohl, Elena Lopez-Rodriguez, Chantal Weissfuss, Jens Kollmeier, Mario Tönnies, Sebastian Brill, Konrad Steinestel, Martin Witzenrath, Werner Wenzel, Christian Zobel*, Geraldine Nouailles*#

*shared, # correspondence: geraldine.nouailles@charite.de

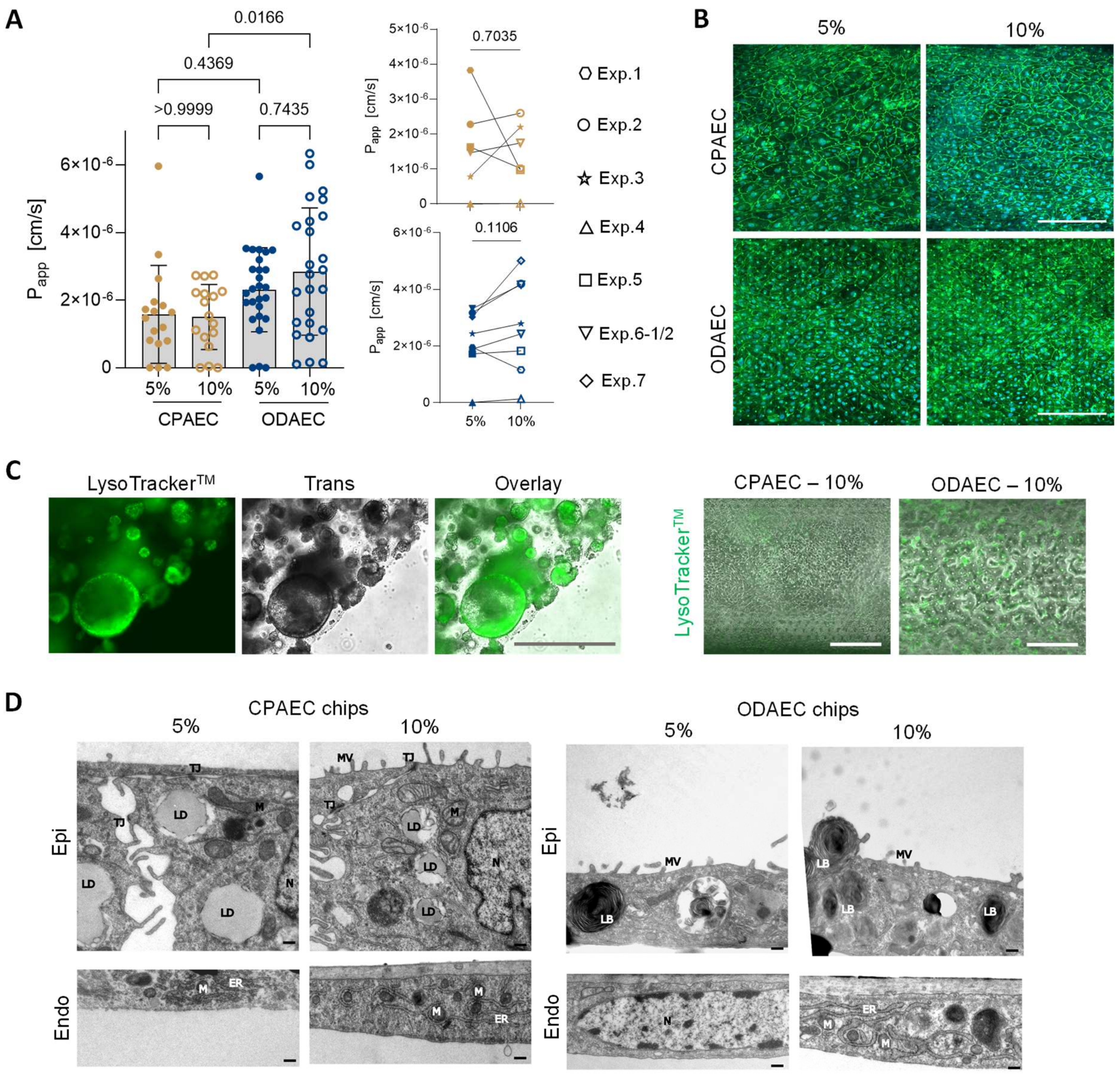

Supplemental Figure S1. CPAECs and ODAECs form a confluent barrier in co-culture with HPMVECs on chip, but only ODAECs produce surfactant. **(A)** The apparent permeability (P_{app}) of CPAECs and ODAECs on chip was measured using a tracer and shows a slightly higher permeability in ODAECs especially under 10% stretch. Left: Bar graph depicts mean ± SEM with data points of all chip replicates over 6-7 independent chip experiments using in total 3 different donors for CPAECs and 7 different donors for ODAECs. While two different ODAEC donors were used in experiment 6 (Exp.6-1/2), the same donor was used in Exp. 4 and 5. One-way ANOVA with Bonferroni's multiple comparison test; $p < 0.05$. Right: Paired line plots depict means of chip replicates of each independent experiment with pairwise comparison; two-tailed paired t tests were performed; $p < 0.05$. **(B)** Representative images of endothelial cells visualized with DAPI (blue) and VE-Cadherin (green), a marker for endothelial adherence junctions, demonstrate the presence of confluent monolayers. Scale bar: 200 µm. **(C)** Exemplary live-cell images of LysoTracker™ treated alveolar organoids (left) and CPAECs and OCAECs seeded on chip (10% stretch, right). Scale bars: 250 µm. **(D)** TEM reveals the presence of lamellar bodies (LB) in ODAECs and lipid droplets (LD) in CPAECs and the formation of tight junctions (TJ). Endothelial cells could be preserved and visualized on the bottom side of the chip. Scale bar: 250 nm. ER = Endoplasmatic reticulum, M = Mitochondrium, MV = Microvilli, N = Nucleus.

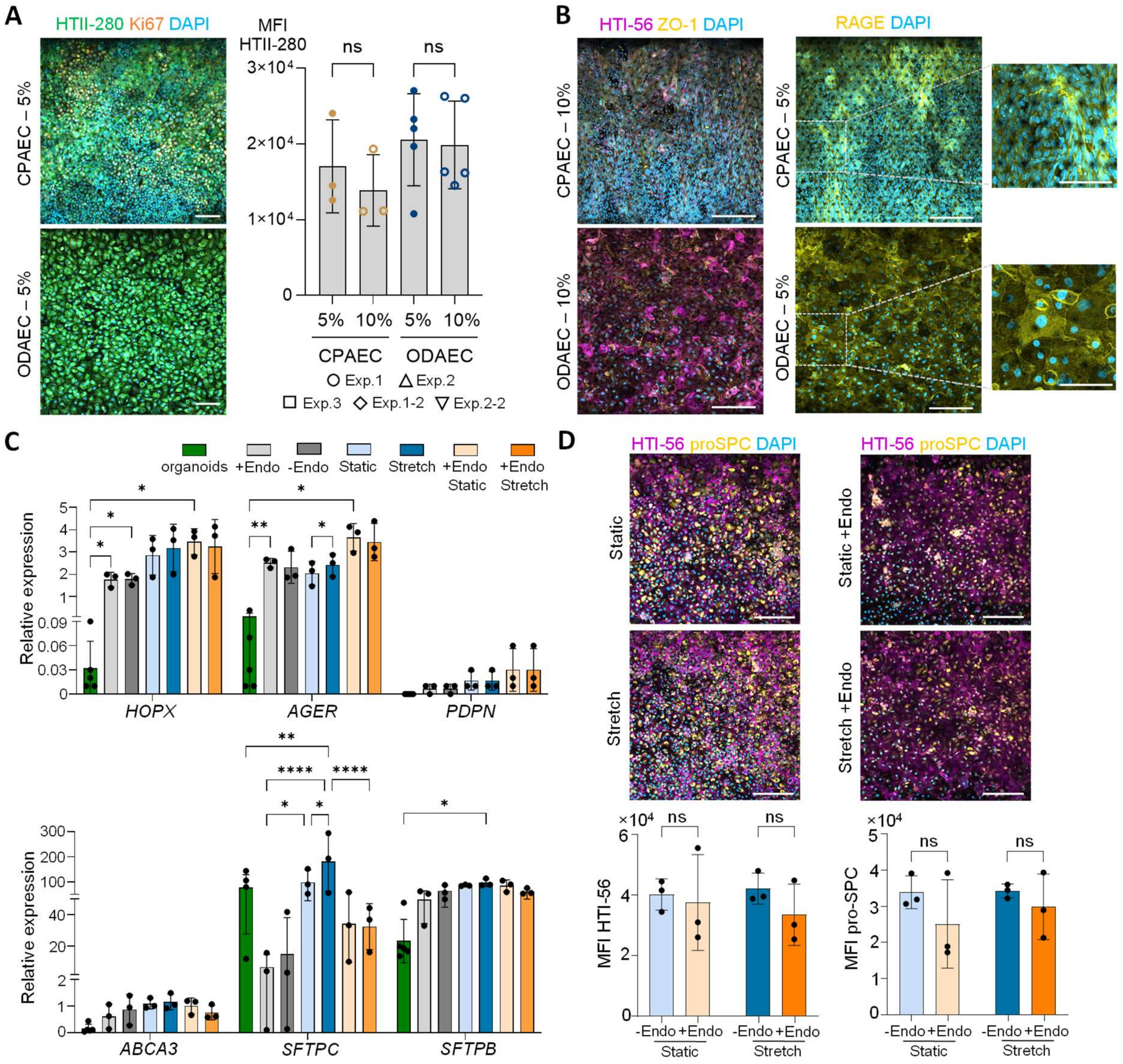

Supplemental Figure S2. CPAECs and ODAECs differ in morphology, proliferation and expression of differentiation marker, while stretch, ALI or the presence of endothelial cells do not have a strong effect on differentiation. (A) Left: HTII-280 (green) and Ki67 (orange) immunostainings of 5% stretched CPAECs and ODAECs. Scale bar: 100 µm. Right: Mean fluorescence intensities (MFI) of HTII-280 levels show an overall slightly lower expression in CPAECs compared to ODAECs and no significant differences between the stretches. Bar graph depicts mean ± SEM of 3 independent experiments with 4 different donors for ODAECs and 3 different donors for CPAECs. One-way ANOVA with Tukey's multiple comparisons test was performed; ns = non significant. **(B)** Left: IF stainings of HTI-56 (magenta) and ZO1 (yellow) in 10% stretched CPAECs and ODAECs reveals clear differences in HTI-56 staining pattern. Right: Immunostainings against RAGE indicate expression in CPAECs is restricted to the cell junctions, while ODAECs also express RAGE on the apical membrane. Scale bars: 200 µm and 50µm. **(C)** Upper graph: qPCR for AT1 marker *HOPX*, *AGER*, and *PDPN* reveals significant differences comparing 2D and the 3D organoid culture, but not within different conditions of the 2D cultures. Lower graph: qPCR for AT2 marker *ABCA3*, *SFTPC*, and *SFTPB.* Mean ± SEM of 3 independent experiments. Two-way ANOVA with Tukey's multiple comparison test, * $p < 0.05$, ** $p < 0.005$, **** $p < 0.00005$. **(D)** Immunostainings of ODAECs against proSPC (yellow) and HTI-56 (magenta) and the calculated MFI (bar graphs). Mean ± SEM of 3 independent experiments is depicted. Two-way ANOVA, ns = non significant. Scale bar: 200 µm.

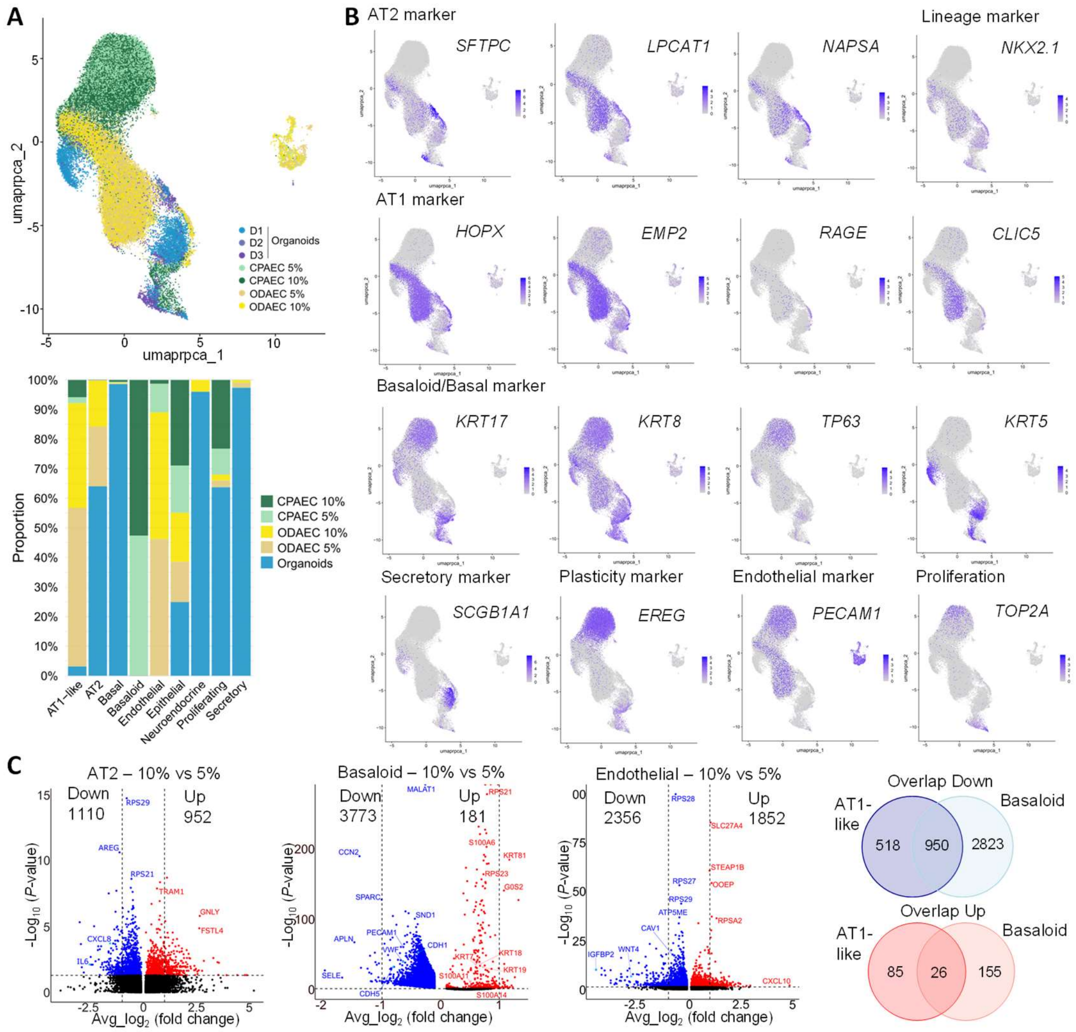

Supplemental Figure S3. Transcriptomic profile of ODAECs and CPAECs. (A) Upper plot: Uniform Manifold Approximation and Projection (UMAP) plot of sequenced and integrated CPAECs, ODAECs and organoids, each indicated with different colors. For the organoids 3 different donors were analyzed (D1, D2 and D3), while for CPAECs and ODAECs only one donor was analyzed but at two different conditions, either 5% or 10% stretch. Lower graph: Bar plot showing the frequencies of annotated cell types for CPAECs, ODAECs and organoids. **(B)** UMAPs displaying specific marker genes as indicated. **(C)** Left: Volcano plots of differentially expressed genes (DEG) between 10% and 5% stretched AT2 cells, Basaloid cells and Endothelial cells. Right: Venn diagrams of overlapping up- or downregulated genes between AT1-like and Basaloid cells.

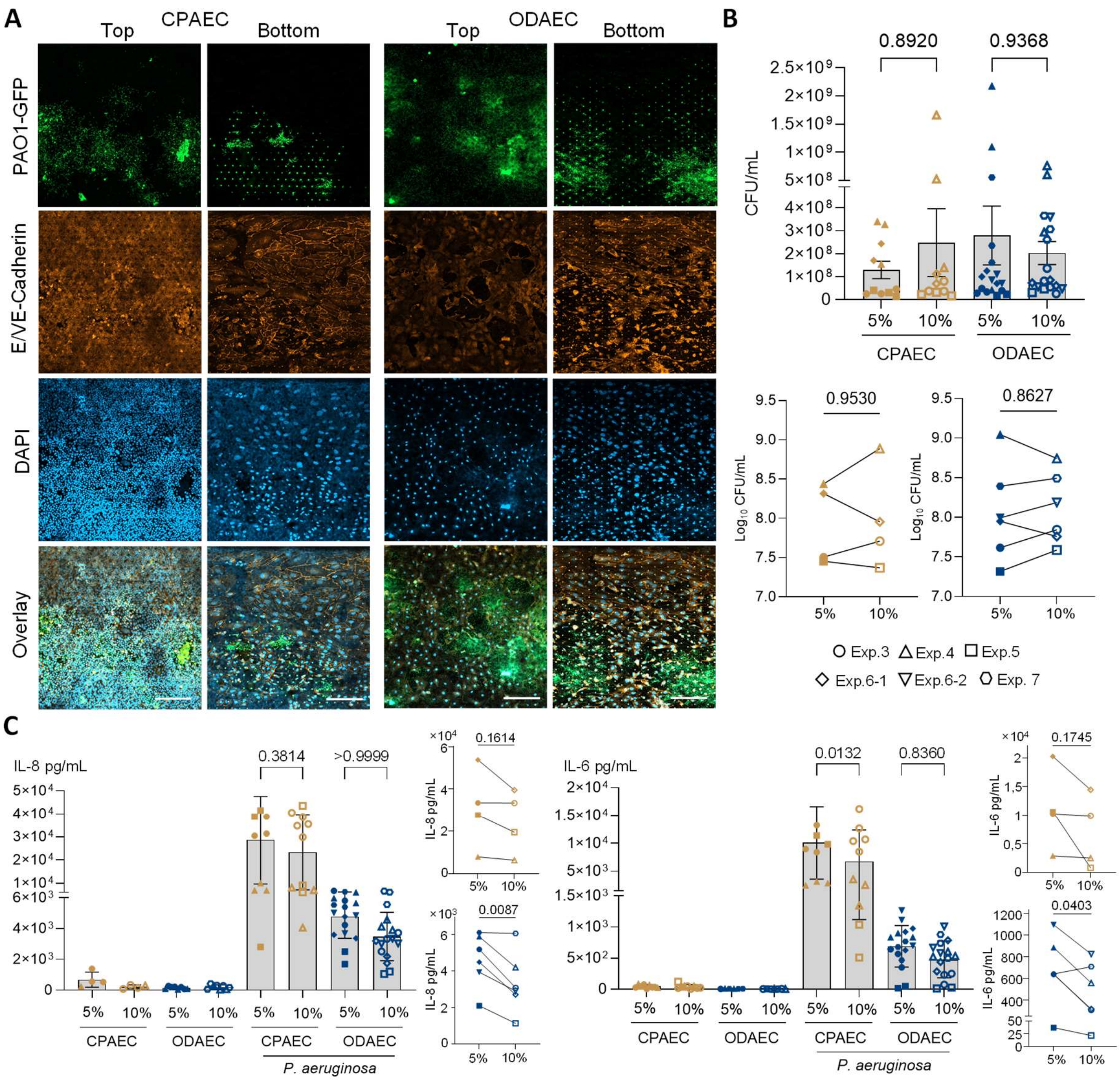

Supplemental Figure S4. Infection of the alveolus chips with *Pseudomonas* induces barrier disruption and a cytokine response. (A) Alveolus chips were infected with *Pseudomonas* strain PAO1-GFP and colonized by the bacteria over a time course of 12 h. Depicted are representative immunostainings of 10% stretched and infected chips showing bacteria (green) on the epithelial (top) and the endothelial (bottom) side and different degrees of disruption of the respective monolayers (E/VE-Cadherin, orange). Scale bars: 200 µm. Disclosure: Close-ups of the overlay images of Bottom CPAEC and ODAEC are also shown in Main Figure 1H. **(B)** Analysis of the bacterial load (CFU/mL) in the epithelial channel shows no significant difference between the different cell types and stretches. **(C)** IL-6 and IL-8 levels determined by ELISA from the vascular effluent of infected (*P.a.*) and non-infected chips at 12 hpi show higher cytokine levels in the CPAEC chips compared to the ODAEC chips and decreased levels in 10% compared to 5% stretched chips. (B and C) Upper/left graphs: Depict mean ± SEM with data points of all chip replicates over 4-6 independent chip experiments using in total 3 different donors for CPAECs and 5 different donors for ODAECs. While two different ODAEC donors were used in experiment 6 (Exp.6-1/2), the same donor was used in Exp. 4 and 5. One-way ANOVA with Bonferroni's multiple comparison test. Lower/right graphs: Paired line plots depict means of chip replicates of each independent experiment with pairwise comparison; two-tailed paired t tests were performed.

Methods

Isolation of epithelial cells and generation of organoids

Macroscopic tumor-free lung resections were processed and alveolar organoids were generated as described previously (1). Altogether 9 different organoid donors were used in 10 independent chip experiments. The primary diagnosis of the donors was either adenocarcinoma or squamous cell carcinoma of the lung. The patients were aged between 56-80 years and of different biological sex (5 male and 4 female).

In short, lung tissue was washed using cold HBSS and chopped using a scissor or scalpel. The minced tissue was collected in an enzyme solution containing HBSS, collagenase I (20 U/µL), Dispase (2 U/mL), DNAse I (50.000 U/mL). The enzyme-tissue mix was incubated in a water bath at 37 °C for 90 min. After vortexing the mixture, it was passed through a sieve and collected in HBSS. Afterward, cells were filtered through a 70 µm filter and centrifuged at 350×g for 7 min. Red Cell Lysis Buffer was added to the pellet and incubated for 5 min at room temperature (RT). After adding 5 mL of Advanced DMEM/F12 supplemented with HEPES (10 mM) and Glutamax (1x) (ADF++), the cells were counted and either stocked or sorted immediately. For sorting the alveolar type 2 (AT2) cell population, freshly isolated cells were stained with LysoTracker™ Green DND-26 (Cell Signaling, 1:10.000) by incubation for 20-30 min at 4°C in the dark in staining buffer (ADF++ with FCS (5%), B27 supplement (1x), N-acetylcysteine (1,25 mM), Nicotinamide (5 mM) and Y-27632 (10 µM)). Cells that were stocked after isolation and thawed again for the cultivation of alveolar organoids were stained with an antibody against surface marker HTII-280 (Terrace, 1:100). Secondary labeling was performed with a donkey anti-mouse IgG (H+L)-AlexaFluor™ 488 or 555 (Thermo Fisher Scientific). Fluorescent-activated cell sorting (FACS) was carried out using the Sony SH800.

The cell pellet obtained after sorting was resuspended in Cultrex RGF Basement Membrane Extract, Type 2 (R&D Systems) at 1000 cells/µL and cultured in the following medium: ADF++ supplemented with R-spondin 1 conditioned medium (10%, conditioned medium was prepared as described previously using the 293T HA Rspo1-Fc cell line (2), B27 supplement (1x), N-acetylcysteine (1,25 mM), Nicotinamide (5 mM), human FGF7 (25 ng/mL), human FGF10 (100 ng/mL), human Noggin (100 ng/mL), A83-01 (0,5 µM), SB202190 (0,5 µM), CHIR99021 (3 µM), human EGF (50 ng/mL), Primocin (1x) and Y-27632 (10 µM). Y-27632 was omitted after 3-5 days. Fresh medium was provided to the organoids every 3-4 days and passaging of organoids was performed every 2-3 weeks at a ratio of 1:2-1:4 by incubation in TrypLE Express (Gibco) for ~6 min at 37°C, followed by washing and resuspension in fresh Cultrex.

Before seeding on the chip or on transwell filters organoids were incubated with LysoTracker™ Green DND-26 (Cell Signaling; 1:10000) to confirm the alveolar phenotype for the majority of the cells. Only in one case organoids had to be purified by sorting for Lysotracker™ positive cells. In general organoids maximum up to passage 6 were used for experiments.

For seeding organoid-derived alveolar epithelial cells (ODAECs), organoids were taken up in cold ADF++, centrifuged (300xg, 5min) and resuspended in TrypLE Express (Gibco) for up to 15 min at 37°C. After enzymatic treatment, organoids were vortexed vigorously and passed 3x through a 27G needle. ODAECs were pelleted, taken up in complete human epithelial cell medium (Cell Biologics, H6621) and counted.

Alveolus-on-a-chip

For generating an Alveolus-on-a-chip model we used the microfluidics organ chip system by Emulate Inc. (Boston, MA, USA). This system allows to mimic the alveolar-capillary interface by seeding epithelial cells on one side and endothelial cells on the other side of a stretchable

membrane. Epithelial air exposure and vascular flow complement the physiological requirements.
The protocol was adapted from Goekeri, C., Linke, K.A.K. *et al.,* 2024 (3). In brief, after activation of the chips with ER1/ER2 components as per manufacturer's instructions, the chips were coated with a mixture of 200 µg/mL collagen IV (Sigma-Aldrich) and 15 µg/mL laminin (Sigma-Aldrich) overnight at 4 °C. On day 0, pre-passaged CPAECs (Cell Biologics, H-6053, Lot # 120718Y31.JS, F101517Y72, F040615Y72NX; all female) or ODAECs (different donors) were seeded on the chip in complete human epithelial cell medium (Cell Biologics, H6621) at a density of 1.6×10^6 cells/mL. Human pulmonary microvascular endothelial cells (HPMECs, Promocell, C-12281, Lot # 480Z005,475Z016 or 463Z013.1; all female) were sequentially seeded on the chip with at least 2 h between seeding of the different cell types. HPMECs were seeded in complete EGM-MV2 (Promocell, C-22121) with a seeding density of 8×10^6 cells/mL. After cell attachment, the chips were washed by gravity flow and kept under static conditions at 37 °C, 5% CO_2. The next day (day 1), the chips were connected to the Pods (Emulate Inc.), and the pods were placed into the Zoe instrument (Emulate Inc.). The instrument was set to perfuse the top and bottom channels of the chips at 40 µL/h. Dexamethasone (100 nM, Sigma-Aldrich, St. Louis) was added to the epithelial cell medium of the top channel to strengthen tight junctions. One to three days later (day 2-4), air-liquid interface culture was induced by flushing the apical epithelial channel at 1,000 µL/h for 1 min. Subsequently, all cells on the chip were only fed from the endothelial channel with EGM-MV2 supplemented with 20 nM Dexamethasone. On day 6, FCS in the EGM-MV2 medium was reduced from 5% to 2%. To mimic physiological breathing at tidal volume the chips were exposed from day 7 or 8 to 5 % cyclic strain at 0.25 Hz (4). As based on previous studies both low-tidal volume (6 mL/kg) and high-tidal volume ventilation (12 mL/kg) can lead to about 10 % of mechanical strain in the alveolus (5-7) half of the chips were exposed to 10% mechanical strain from day 9 or 10 until day 14.
For live cell staining with LysoTracker™ Green DND-26 (Cell Signaling), the LysoTracker™ was diluted in the vascular medium at 1:10000 and the chips fed with the LysoTracker™ medium overnight. The next day chips were imaged using the Revolve (ECHO) or the LSM980 (ZEISS) microscope. Afterwards vascular medium without LysoTracker™ was added to the pods.

Transwell cultures

ODAECs were seeded on transwell filters (CellQart, 9323012, 24-well inserts with 3µm PET clear) as described for the chip device (see above). First, the filters were coated with a mixture of 200 µg/mL collagen IV (Sigma-Aldrich) and 15 µg/mL laminin (Sigma-Aldrich) overnight at 4 °C and then the organoid-derived cells were seeded in complete human epithelial cell medium (Cell Biologics, H6621) at a density of $\sim 0{,}5 \times 10^6$ cells/mL. Using a volume of 100 µl resulted in similar cell numbers as seeded on the chip device. Also, in line with the alveolus-chip protocol cell were fed from the bottom chamber with the endothelial EGM-MV2 medium (Promocell, C-22121). The cells were either kept in liquid-liquid interface (LLI) or lifted to air-liquid interface (ALI) after reaching confluence at ~2 days post-seeding. After 2 weeks the LLI and ALI transwell cultures were terminated in parallel with the chip cultures by taking RNA samples.

Apparent permeability (P_{app}) testing

Barrier function was analyzed by applying tracer (C687 Cascade Blue™ hydrazide, Trisodium Salt, Invitrogen, Waltham, MA, USA) from the bottom endothelial channel for 2 h (160 µL/h)

and collecting outflows from the apical epithelial channel as well as the bottom channel, either at least 12 h prior infection or 12 h post-infection (hpi). Fluorescence was measured with a SpectraMax M2e plate reader (Molecular Devices, San Jose, CA, USA), and apparent permeability (P_{app}) was calculated as described previously (8).

Bacterial culture and infection of the alveolus-chips

Pseudomonas aeruginosa strain PAO1 tagged with a GFP (PAO1-GFP) was streaked out on blood agar plates from a glycerol stock the day before infection. On the day of infection colonies were picked from the blood agar plates, added to 5 mL of TSB Medium (in a 50 mL tube), and adjusted to OD = 0,05 - 0,07. The bacterial suspension was incubated for about ~2 h at 37 °C, 220 rpm. Subsequently, bacteria were pelleted (3500 rpm, 10 min, RT), washed twice with 10 mL pre-warmed PBS, and resuspended in 5-10 mL PBS. The OD was measured based on a McFarland standard and the suspension was adjusted to 1.5×10^8 CFU/mL in PBS. This suspension was further diluted to a calculated concentration of 1 CFU/ µL in basal epithelial medium (Cell biologics, M6621b) without any supplements. The actual bacterial concentration was determined by streaking out 50 µL of the diluted bacterial suspension on a blood agar plate revealing for actual concentrations of 3-10 CFUs/µL.

Chips with an intact barrier (no leakage in the apical channel) were used on day 15 for infection. At least 24h prior infection, the medium was removed from pods, pods were washed with 2mL PBS and replenished with antibiotic-free medium. For infection, the chips were disconnected from the pods and 40 µL of bacterial suspension (3-10 CFU/µL) were introduced by gravity flow into the top channel (epithelial side). The bacteria were incubated on the chip for 1h at 37°C, 5% CO_2. After incubation pipette tips were removed and chips were re-connected to the pods and ZOE. A flush cycle (1000 µL/h) was performed for 1 min to re-introduce ALI in the top channel. Chips were incubated under stretch and flow and endpoint analysis was performed at 12 hpi.

Bacterial burden

At 12 hpi bacterial burden in the vascular and the epithelial compartment was determined by first collecting medium from the vascular flow-through and streaking out serial dilutions from 10^0-10^{-4}. Next, tracer analysis was performed and after 2 h flow (160 µL/h) the flow-through from the epithelial side was collected to determine the bacterial burden. Serial dilutions were prepared (10^{-1}-10^{-7}) and streaked on blood agar plates. Colonies were counted after overnight (ON) incubation at 37°C, 5% CO_2.

Immunofluorescence stainings

For immunofluorescence staining, chips were either cut in half or stained as a whole after fixation with 3.7% paraformaldehyde for 20 min at room temperature (RT). After fixation, chips were washed twice with 1x PBS, permeabilized for 30 min at RT (0,1% Triton-X-100), blocked for 2 h at RT (10% donkey serum in 1% BSA/PBS), and washed with 200 µL 1xPBS. Primary antibodies were diluted in 1% BSA/PBS and incubated at 4 °C ON. The bottom channel was perfused with mouse anti-VE-Cadherin antibody (1:100, sc9989, Santa Cruz Biotechnology) to stain endothelial junctions and GFP Polyclonal Antibody with Alexa Fluor™ 488 (1:100, A-21311, Thermo Fisher Scientific) to label the bacteria, while the top channel was exposed to the indicated antibodies: mouse anti-HTI-56 antibody (1:200, TB-29AHT1-56, Terrace Biotech), mouse anti-HTII-280 antibody (1:200, TB-27AHT2-280, Terrace Biotech), rabbit anti-ZO-1 antibody (1:100, PA5-28858, Thermo Fisher Scientific), rabbit anti-GFP polyclonal antibody with Alexa Fluor™ 488 (1:100, A-21311, Thermo Fisher Scientific), rabbit anti-

Prosurfactant Protein C (proSP-C) antibody (1:1000, AB3786, Merck), rabbit anti-Ki-67 (D2H10) antibody (1:100, 9027, Cell signaling) and goat anti-RAGE antibody (1:200, AF1179-SP, R&D Systems). After washing with 200 µL 1xPBS, chips were incubated with secondary antibodies (1:1000, A-21202, donkey anti-mouse IgG Alexa Fluor™ 488; A-31572 or donkey anti-Rabbit IgG Alexa Fluor™ 555, Thermo Fisher Scientific) for 2 h at RT. Washing was followed by incubation with DAPI (ab228549, Abcam) for 15 min at RT and final washing (200 µL 1xPBS). Stained chips were analyzed with an LSM980 confocal microscope (ZEISS).

Cytokine measurements
Supernatants were collected from the vascular flow-through of the chip pre- and post-infection. Cytokines IL-6 and IL-8 were measured by performing an Enzyme-linked immunosorbent assay (ELISA) using the BD OptEIA™ Kits (BD Biosciences 555220 and 555244). ELISAs were performed according to the manufacturer's instructions.

RNA isolation and qPCR
RNA was isolated from chips using the RNeasy Plus Micro Kit (Qiagen, 74034) and lysing the cells directly on the chip. RNA from organoids was isolated with the RNeasy Mini Kit (Qiagen, 74104).
For quantitative real-time PCR (qRT-PCR) the RNA was diluted to 2 ng/µL and reverse transcription was performed using the High-Capacity cDNA Reverse Transcription Kit (Thermo Fisher Scientific, 4368813) according to manufacturer instructions. Gene expression was measured using the TaqMan™ Fast Advanced Master Mix (Thermo Fisher Scientific, 4444556) using the QIAquant 96 5plex cycler (Qiagen, program: 1. Hold, 95°C, 20 sec; 2. 40 cycles: 95°C, 1 sec and 60°C, 20 sec) and the following TaqMan® Assays (Thermo Fisher Scientific): Hs05028646_s1 (*HOPX*), Hs00366766_m1 (*Podoplanin*), Hs00184543_m1 (*ABCA3*), Hs00161628_m1 (*SFTPC*), Hs00167036_m1 (*SFTPB*), Hs00971716_m1 (*CAV1*), Hs02786624_g1 (*GAPDH*), Hs00542584_g1 (*RAGE*).

Transmission electron microscopy
The lung-on-a-chip cell membranes were fixed in a fixative solution containing 1.5% PFA with 1.5% GA in 0.15 M HEPES buffer.
The membranes were carefully extracted from the chip and embedded in a flat orientation in Epon as follows. The membranes were post-fixed using 1% OsO4 (Electron Microscopy Sciences) in 0.1 M cacodylate buffer (Serva) at room temperature (RT) for 2 h, followed by incubation in half-saturated aqueous uranyl acetate (Serva) overnight at 4°C. After dehydration in a graded acetone series (Roth), membranes were transferred to Epon resin (Serva). Finally, ultrathin sections of 70 nm were cut using an ultramicrotome (Leica) and a diamond knife (Diatome), collected on pioloform-coated copper grids (Plano), and stained with lead citrate (Merck Millipore) according to Reynolds (Reynolds, 1963). The examination of the grids was performed with a Zeiss Leo 906 electron microscope (Carl Zeiss) at 80 kV acceleration voltage equipped with a slow scan 2 K charge-coupled device (CCD) camera (TRS-Tröndle) with associated software Image SP (version 1.2.890).

Single cell transcriptomics
Viable cells from 4 experimental groups (CPAECs and ODAECs either 5% or 10% stretched) were used for single cell RNA sequencing (Sc-Seq) according to the Chromium Next GEM Single Cell 3', v.3.1 workflow (10x Genomics, Pleasanton, CA, USA). GEM generation, cDNA amplification and library construction were performed according to manufacturer’s instructions.

Amplified libraries were quality controlled by Qubit fluorometric quantification with the dsDNA HS assay kit (Invitrogen, USA) and Bioanalyzer using the High Sensitivity DNA Kit (Agilent Technologies, USA) prior sequencing according to the Illumina protocol on a NovaSeq X Series device using recommended sequencing conditions.
Sequencing data processing was performed using nf-core/scrnaseq pipeline v.3.18.0 (9) with CellRanger aligner, 10XV3 protocol, and Singularity containers as well as background correction using cellbender 0.3.2. This also included pre-processing of output files using cell ranger functions count and mkfastq (10x Genomics Cell Ranger Software, Pleasanton, CA, USA). Downstream analyses were performed using software R version 4.4.0 (Vienna, Austria) including packages Seurat (v5) (10), scDblFinder for initial doublet detection, and demuxafy 3.0.0. (including packages solo, scDblFinder, scds, and scrublet) for comprehensive doublet detection (11). Multiple doublet detection methods were compared, with consensus doublets (≥2 methods) flagged for removal after initial filtering regarding scDblFinder. Low-quality cells were also removed if cells showed less than 100 different genes or more than 10 000, less than 100 RNA counts, or more than 100 000 or more than 30% mitochondrial RNAs. When cells were clustered using standard Seurat workflow of the combined data with PCA reduction (30 dimensions) and graph-based clustering at resolution 0.5), clusters with more than 50% low-quality cells were removed. Resulting data of the four experimental groups were integrated with RPCA implemented in Seurat v5, after testing multiple methods including RPCA, Harmony, scVI, FastMNN, Joint PCA, and CCA integration. Resulting integrated clusters were again quality controlled excluding a cluster with >50% poor-quality cells. The generated data sets were furthermore integrated with a previously published Sc-Seq data set of alveolar organoids (1), again with RPCA using Seurat v5. Clusters were assigned to cell types applying GPTCelltype (12) followed by manual curation based on marker gene expression including the following: AT2 cells (*SFTPC*, *SFTPB*, *LPCAT1*, *NAPSA*, and *ABCA3*), AT1 cells (*HOPX*, *EMP2*, *CLIC5*, *ITGB6*, *RAGE*, and *PDPN*), Basaloid cells (*KRT17*, *KRT8*, *KRT18*, *CLDN4*, *TP63*), Basal cells (*KRT5*), Secretory cells (*SCGB3A2*, *SCGB1A1*, *SCGB3A1*), Proliferative cells (*TOP2A*, *PCNA*, and *MKI67*), Endothelial cells (*PECAM1*, *CAV1*, and *CDH5*).
Differential expression was performed using a Wilcoxon rank-sum test and *P* values were adjusted for multiple testing using the Bonferroni correction.

Sex as a biological variable
The human cells used in this study were of female or male origin (see above)). However, sex was not considered as a biological variable in this study.

Statistics and reproducibility
Data analysis was carried out if not stated otherwise using GraphPad Prism 10 (GraphPad Software). Statistical significance was determined as described in the figure legends. A p-value less than 0.05 was considered statistically significant. When comparing more than two groups appropriate correction for multiple comparisons was performed. All experiments were repeated at least three times with 2-3 chip replicates and unless stated otherwise data are represented as the mean ± SEM with either individual chip replicates or the means of the independent experiments being depicted as data points. For each cell type (CPAECs, ODAECs, and HPMVECs) at least three different donors were tested. The microscopy images provided are representative of consistent observations across multiple fields. Mean fluorescence intensities (MFI) were always derived from at least six IF images of each independent chip experiment with at least three independent chip experiments being performed using at least three different donors.

Graphics
The schematic in Figure 1A was created in BioRender. Nouailles, G. (2026) https://BioRender.com/g4yc5m2.

Study approval
Human lung resection samples were provided by the Institute of Pathology of the Bundeswehr hospital Ulm or by the Emil-von-Behring Hospital Berlin after informed patient consent. The study has been approved by the Ethics Committee of the Berliner Ärztekammer under the ethics approval number Eth-03/23.

Data availability
Single-cell RNA sequencing data files are publicly available through Zenodo via https://doi.org/10.5281/zenodo.17041981; the code used for data analysis is available at github.com, https://github.com/GenStatLeipzig/2412_scRNA_lung_organoid_chip.

Acknowledgments
We thank Jean-Marc Ghigo from the Institute Pasteur and a member of the "MAPVAP" consortium for kindly providing the PAO1-GFP strain to our lab. We thank Virginia Weber, Annette Daniel, Ute Mauer, and John Horn for their valuable technical support. During the preparation of this work, the authors used ChatGPT / OpenAI, Grammarly / Grammarly, Inc. to improve English language and grammar, as well as German to English translations. After using these tools, the authors reviewed and edited the content as needed and take full responsibility for the content of the publication.
This study was funded by the Department E of the Bundeswehr Medical Service Academy (SoFo 39K4), Ministry of Defence, Germany, and BAAINBw (E/U2ED/PD014/OF550) and the Deutsche Forschungsgemeinschaft (DFG, German Research Foundation) – Project ID 431232613 – SFB 1449. HK received funding from the Federal Ministry of Research, Technology and Space (BMFTR) within the project "Center for Scalable Data Analytics and Artificial Intelligence (ScaDS.AI) Dresden/Leipzig" (grant 01IS18026B).

References
1. Hoffmann K, Obermayer B, Honzke K, Fatykhova D, Demir Z, Lowa A, et al. Human alveolar progenitors generate dual lineage bronchioalveolar organoids. *Commun Biol.* 2022;5(1):875.
2. Hoffmann K, Berger H, Kulbe H, Thillainadarasan S, Mollenkopf HJ, Zemojtel T, et al. Stable expansion of high-grade serous ovarian cancer organoids requires a low-Wnt environment. *EMBO J.* 2020;39(6):e104013.
3. Goekeri C, Linke KAK, Hoffmann K, Lopez-Rodriguez E, Gluhovic V, Voss A, et al. Enzymatic Modulation of the Pulmonary Glycocalyx Enhances Susceptibility to Streptococcus pneumoniae. *Am J Respir Cell Mol Biol.* 2024;71(6):646–58.
4. Huh D, Matthews BD, Mammoto A, Montoya-Zavala M, Hsin HY, and Ingber DE. Reconstituting organ-level lung functions on a chip. *Science.* 2010;328(5986):1662–8.
5. Knudsen L, and Ochs M. The micromechanics of lung alveoli: structure and function of surfactant and tissue components. *Histochem Cell Biol.* 2018;150(6):661–76.
6. Xie J, Jin F, Pan C, Liu S, Liu L, Xu J, et al. The effects of low tidal ventilation on lung strain correlate with respiratory system compliance. *Crit Care.* 2017;21(1):23.

7. Waters CM, Roan E, and Navajas D. Mechanobiology in lung epithelial cells: measurements, perturbations, and responses. *Compr Physiol.* 2012;2(1):1–29.
8. Si L, Bai H, Rodas M, Cao W, Oh CY, Jiang A, et al. A human-airway-on-a-chip for the rapid identification of candidate antiviral therapeutics and prophylactics. *Nat Biomed Eng.* 2021;5(8):815–29.
9. 10.5281/zenodo.1400710.
10. Hao Y, Stuart T, Kowalski MH, Choudhary S, Hoffman P, Hartman A, et al. Dictionary learning for integrative, multimodal and scalable single-cell analysis. *Nat Biotechnol.* 2024;42(2):293–304.
11. Neavin D, Senabouth A, Arora H, Lee JTH, Ripoll-Cladellas A, sc-e QC, et al. Demuxafy: improvement in droplet assignment by integrating multiple single-cell demultiplexing and doublet detection methods. *Genome Biol.* 2024;25(1):94.
12. Hou W, and Ji Z. Assessing GPT-4 for cell type annotation in single-cell RNA-seq analysis. *Nat Methods.* 2024;21(8):1462–5.
